# The Treatment of Heterotopic Human Colon Xenograft Tumors in Mice with 5-Fluorouracil Attached to Magnetic Nanoparticles in Combination with Magnetic Hyperthermia Is More Efficient than Either Therapy Alone

**DOI:** 10.3390/cancers12092562

**Published:** 2020-09-09

**Authors:** Mohammad Dabaghi, Rainer Quaas, Ingrid Hilger

**Affiliations:** 1 Department of Experimental Radiology, Institute for Diagnostic and Interventional Radiology, Jena University Hospital—Friedrich Schiller University Jena, D-07747 Jena, Germany; mohammad.dabaghi@med.uni-jena.de; 2 Chemicell GmbH, Erseburgstrasse 22-23, 12103 Berlin, Germany; quaas@chemicell.com

**Keywords:** colorectal cancer, magnetic hyperthermia, iron oxide nanoparticles, 5-fluorouracil

## Abstract

**Simple Summary:**

In spite of considerable advancements in cancer treatment, there is still a high incidence and mortality of colorectal cancer. The limited effectiveness of current therapies demands innovative and more promising therapeutic strategies. In this study, we therefore examined the potency of a combined therapeutic approach employing magnetic nanoparticles for the induction of magnetic hyperthermia and a 5-fluorouracil-based chemotherapy, termed thermo-chemotherapy. The main objective was to enable the relapse-free eradication of colorectal cancer. In mice models of colorectal cancer, we found successful reduction of the proliferation potential, extensive DNA damage in the treated tumor cells, profound damage to tumor vascular system, and reduced tumor volume, induced by our local combinatorial thermo-chemotherapy. These findings expose potential benefits of the thermo-chemotherapy approach for the effective treatment of colorectal cancer in the future.

**Abstract:**

Magnetic nanoparticles (MNPs) have shown promising features to be utilized in combinatorial magnetic hyperthermia and chemotherapy. Here, we assessed if a thermo-chemotherapeutic approach consisting of the intratumoral application of functionalized chitosan-coated MNPs (CS-MNPs) with 5-fluorouracil (5FU) and magnetic hyperthermia prospectively improves the treatment of colorectal cancer. With utilization of a human colorectal cancer (HT29) heterotopic tumor model in mice, we showed that the thermo-chemotherapeutic treatment is more efficient in inactivating colon cancer than either tumor treatments alone (i.e., magnetic hyperthermia vs. the presence of 5FU attached to MNPs). In particular, the thermo-chemotherapeutic treatment significantly (*p* < 0.01) impacts tumor volume and tumor cell proliferation (Ki67 expression, *p* < 0.001) compared to the single therapy modalities. The thermo-chemotherapeutic treatment: (a) affects DNA replication and repair as measured by H2AX and phosphorylated H2AX expression (*p* < 0.05 to 0.001), (b) it does not distinctly induce apoptosis nor necroptosis in target cells, since expression of p53, PARP cleaved-PARP, caspases and phosphorylated-RIP3 was non-conspicuous, (c) it renders tumor cells surviving therapy more sensitive to further therapy sessions as indicated by an increased expression of p53, reduced expression of NF-κB and HSPs, albeit by tendency with *p* > 0.05), and (d) that it impacts tumor vascularity (reduced expression of CD31 and αvβ3 integrin (*p* < 0.01 to 0.001) and consequently nutrient supply to tumors. We further hypothesize that tumor cells die, at least in parts, via a ROS dependent mechanism called oxeiptosis. Taken together, a very effective elimination of colon cancers seems to be feasible by utilization of repeated thermo-chemotherapeutic therapy sessions in the long-term.

## 1. Introduction

Colorectal cancer is the third most common and second deadliest cancer worldwide, which led to approximately 881,000 deaths in 2018 [1]. Colorectal cancers mostly develop from non-cancerous polyps over a period of 10 to 15 years [2]. Particularly in earlier stages and subsequent to adjuvant therapy, such as chemotherapy and radiation therapy, surgery is one of the standard treatments for this cancer entity. Among the many available chemotherapeutic drugs, 5-fluorouracil (5FU) is widely used. Namely, 5FU is an analogue of uracil that rapidly enters the cell and converts itself into active metabolites, such as fluorodeoxyuridine monophosphates, fluorodeoxyuridine triphosphates, and fluorouridine triphosphates [3]. These intracellularly active 5FU metabolites inhibit the activity of thymidylate synthase and interfere with RNA and DNA synthesis. Thymidylate synthase is an important enzyme for pyrimidine synthesis, and pyrimidine is crucial for DNA replication. Unfortunately, a monotherapy of colorectal cancer with 5FU is not highly effective (approximately 10–20% response rates), and an increase in dose would lead to adverse side effects and drug resistance [4]. Interestingly, the combination of 5FU with other chemotherapeutic drugs, such as oxaliplatin, has improved the tumor therapeutic outcomes [5]. However, further therapeutic strategy developments seem to be urgently needed in order to decrease adverse side effects during treatment.

Combining hyperthermia and chemotherapy as a therapy option has recently gained the attention of researchers and oncologists. In cancer therapy, hyperthermia is defined to be the treatment of a tumor with a temporary rise of temperatures (≥43 °C, 60 min for oncological purposes) [6,7]. Basically, the application of the required temperatures can be performed locally, regionally, and in the whole body using different techniques [8]. Previous studies have confirmed that a temperature increase up to 39 and 42 °C for 30 to 360 min leads to tumor cell death by necrosis and/or apoptosis [6,9,10]. With particular consideration of colon carcinomas, mild hyperthermia tumor treatments (e.g., 41.5 °C for 2 h) have been reported to significantly delay tumor growth [9]. It has further been shown that hyperthermia has an impact on the cytotoxic potential of various anti-cancer drugs, due to an effect called “thermal chemosensitization” [6].

Combining regional radiofrequency-based hyperthermia, which has an external heat source, with radiotherapy and chemotherapy to treat colorectal cancer has shown promising therapeutic results [11]. However, when using an external heat source, it is challenging and it requires adequate tools to properly focus the delivered energy and raise the temperature only on the target tumor tissue. On the contrary, internal heating sources have the advantage of facilitating a highly localized energy deposition within the tumor region. In this respect, and with regard to the combination of hyperthermia with standard oncological treatment modalities, magnetic hyperthermia has recently become increasingly the focus of research. In principle, tissue internal heating sources can be deposited by utilization of various magnetic materials, such as iron oxide magnetic nanoparticles (MNP) [12]. Such materials can be deposited by injection into tumors. They transiently generate heating when exposed to an alternating magnetic field (AMF) [13]. MNPs used in magnetic hyperthermia applications typically have a single or multi-domain magnetite core (e.g., iron oxide) coated with an organic and/or inorganic material. The appropriate selection of MNPs with a high specific absorption rate (SAR) to generate the required heat and their potential for coupling them with anticancer drugs for local delivery are key factors to effectively kill proliferating tumor cells [6,14]. Several studies used different combinations of magnetic hyperthermia with various chemotherapeutic agents (e.g., doxorubicin and methotrexate) after having attached them to the coating material (mostly a polymer) on the surface of MNP for the treatment of various cancers in mice, such as breast and bladder [15,16]. 

Interestingly, several studies used chitosan-coated MNPs (CS-MNPs) to encapsulate 5FU for drug delivery applications as a single therapeutic modality [17,18] or in combination with hyperthermia [19,20]. Further on, it has been reported that the combination of 5FU with magnetic hyperthermia leads to a reduced viability and proliferation behavior of colon cancer cells in vitro [19,20]. However, since these studies were carried out mostly in vitro as proof of principles, further in vivo investigations are essential. 

In this study we sought to analyze if the thermo-chemotherapeutic approach composed of the intratumoral application of 5FU attached to MNPs and magnetic hyperthermia is more efficient in inactivating colon cancer than either tumor treatment alone (magnetic hyperthermia vs. the presence of 5FU attached to MNPs). In this context, we analyzed to which extent the thermo-chemotherapeutic treatment impacts tumor volume and tumor cell proliferation (Ki67 expression) compared to the single therapy modalities. We further assessed to which extent the thermo-chemotherapeutic treatment: (a) affects DNA replication and repair (presence of H2AX and phosphorylated H2AX) in target cells, (b) it induces apoptosis in target cells (presence of p53, PARP cleaved PARP, and of caspases), (c) it renders tumor cells surviving therapy prospectively more sensitive to further therapy sessions (presence of p53, NF-κB, HSPs), and (d) how it impacts tumor vascularity (presence of CD31 and αvβ3) and consequently nutrient supply. We further discuss the possible mechanism of cell death as result of the thermo-chemotherapeutic approach. 

## 2. Results

### 2.1. Impact of the 5 FU Functionalization on the Morphological Features of CS-MNPs

Chitosan coated MNPs (CS-MNPs) were functionalized with 5-fluorouracil via inter-molecular hydrogen bonding interactions between hydroxide (OH) in chitosan molecule and oxygen (O) in 5FU molecule (5FU, see methods). The pH of CS-MNP increased from 7 to 7.5 after functionalization with 5FU. UV-spectroscopy analysis used to calculate the concentration of 5FU and confirmed that 5FU molecules were coupled to CS-MNPs. The concentration of coupled 5FU was 138 ± 14 µg/mg Fe (*n* = 5).

The size notably increased when 5FU molecules were coupled with the CS-MNPs (named as 5FU-CS-MNP) compared to their non-coupled counterpart. The polydispersity indexes (PdI) were almost comparable between both MNP groups, and there was a drastic reduction of ζ-potential of CS-MNP after functionalization with 5FU. The iron content of both 5FU-CS-MNP and CS-MNP groups were almost comparable, and the SAR of the MNPs did not considerably change after functionalization with 5FU (Table 1).

### 2.2. Control and Monitoring the Tumor and Body Temperatures of Tumor Bearing Mice

To assess the effect of the thermo-chemotherapeutic colon cancer treatment (5FU-MNP-based chemotherapy and magnetic hyperthermia) animal experiments with heterotopic colon carcinoma (HT29 human carcinoma cells) bearing mice were performed. Hereto, six independent animal groups were defined as follows: (a) the combinatorial treatment group (5FU-MNP/MH), (b) the magnetic hyperthermia treatment alone group (MNP/MH), (c) the 5FU-MNP-based chemotherapy alone group (5FU-MNP), (d) the MNP injected group (MNP), (e) 5FU injected group (5FU, for details see methods), and (f) the non-treated group. After intratumoral injection of the 5FU-CS-MNPs, two hyperthermia sessions with a time interval of six days were undertaken (for details see methods). 

**Figure 1:**
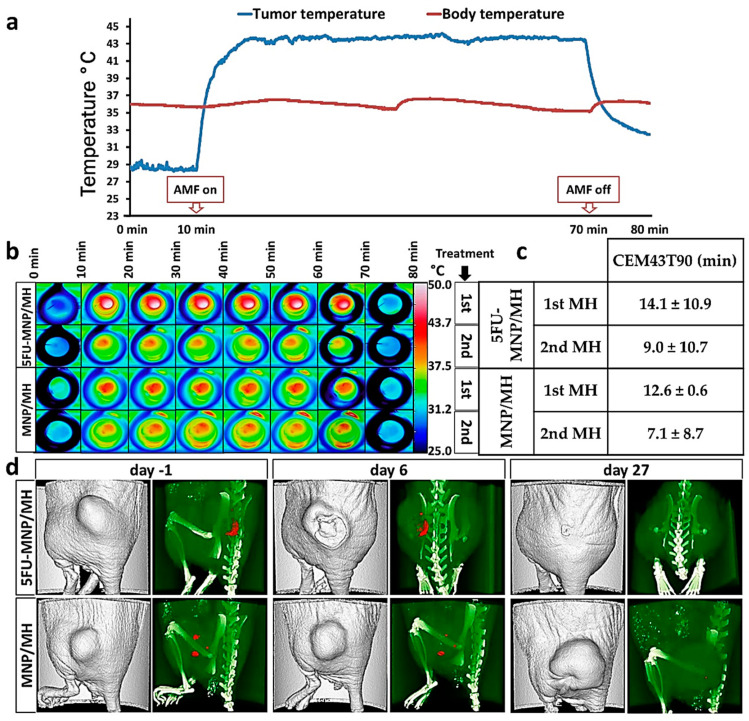
Monitoring the real-time temperature of the tumor and body led to an effective application of magnetic hyperthermia. (**a**) Representative diagram of tumor and rectal temperatures after MNP injection (CS-MNP and 5FU-CS-MNP, 0.25 mg Fe per 100 mm^3^ tumor tissue and 0.22 to 0.55 mg 5FU per kg body weight) and exposure to an AMF (60 min at *H* = 15.4 kA/m, *f* = 410 kHz), (**b**) heat maps of the tumor surface temperatures as recorded with a thermographic camera, (**c**) mean CEM43T90, calculated for magnetic hyperthermia treatments and (**d**) representative µCT overlay images (bones: white, MNPs: red) taken at 24 h before the first (day −1) and second tumor therapy (day 6) and 24 h before the animal sacrifice (day 27) showing the clearance of MNPs over time. AMF: alternating magnetic field; MH: magnetic hyperthermia; µCT, micro computed tomography; MNP; magnetic nanoparticle; 5FU: 5-fluorouracil; CEM: cumulative equivalent minutes.

The recorded temperatures during treatment of mice, which have been allocated either to the combinatorial treatment group (5FU-MNP/MH) or the magnetic hyperthermia treatment alone group (MNP/MH), indicated that the tumor temperature started to increase once the AMF was switched on and dropped when it was off, while the body temperature remained almost constant during the treatment (Figure 1a,b). In general, the tumor temperature dose of animals allocated to the 5FU-MNP/MH group was slightly higher, but not significantly different (with *p* > 0.05) compared to those of the magnetic hyperthermia only group (Figure 1c). The temperature dose was estimated according to Sapareto and Dewey 1984 [10] whereby given temperature-time curves are converted into an equivalent treatment time at 43 °C, and where the 90th percentile of the temperatures measured at the tumor surface are calculated (CEM43T90, for details see methods). Micro computed tomography (µCT) imaging confirmed that the MNPs were heterogeneously distributed within the tumor, and that they were completely or partially cleared away over time along with the elimination of the treated part of the tumor (Figure 1d). 

### 2.3. The Thermo-Chemotherapeutic Tumor Treatment Significantly Reduces the Tumor Volume

**Figure 2:**
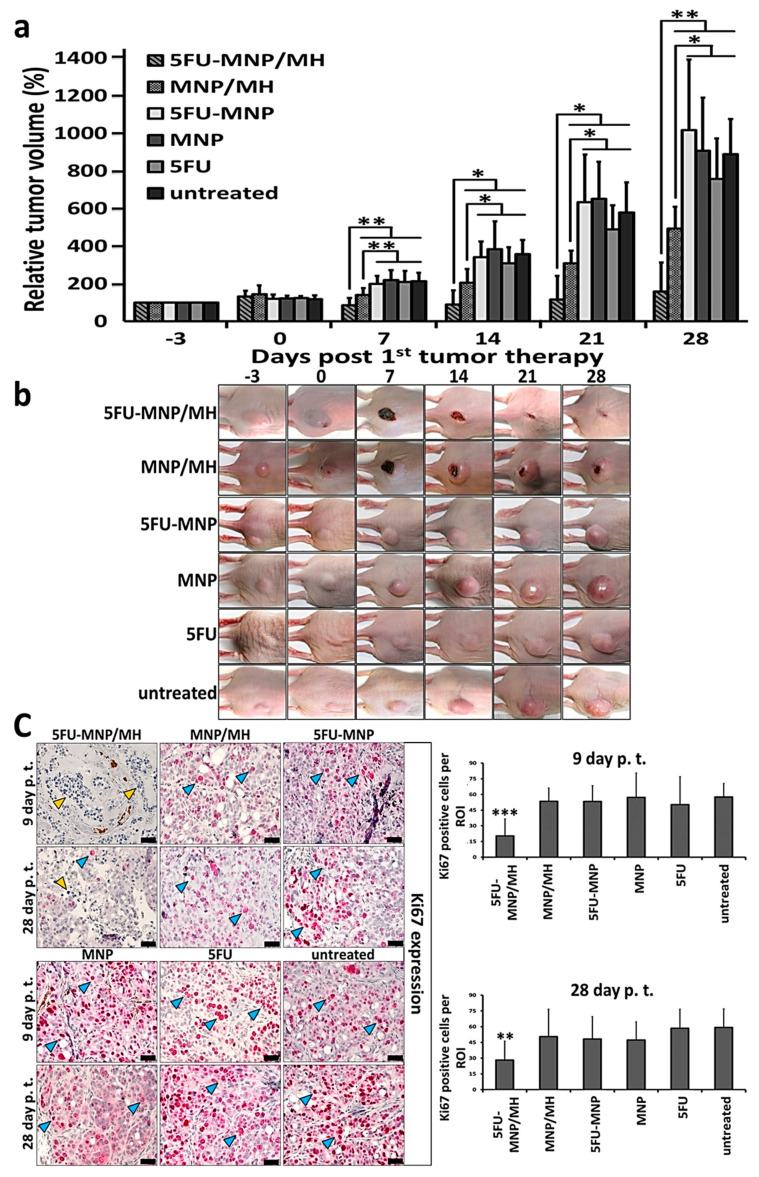
The combinatorial thermo-chemotherapeutic tumor treatment resulted in a significant tumor volume reduction (* *p* < 0.05 for days 14 and 21 post first tumor therapy and ** *p* < 0.01 for days 7 and 28 post first tumor therapy) compared to all other treatments. (**a**) Development of tumor volume over time in the combinatorial treatment (5FU-MNP/MH; 60 min at *H* = 15.4 kA/m, *f* = 410 kHz) and control groups. Tumor volume in percentage was calculated relative to the tumor volume on day −3 post first tumor therapy. (**b**) Representative photographs of tumors in each group during therapy. (**c**) Reduction of Ki67 expression in tumors treated with the combinatorial therapy (5FU-MNP/MH) compared to the either therapies alone (5FU-MNP or MH). Depicted are representative histological micrographs of tumor tissue slices after immunohistochemical staining as well as semi-quantitative estimations of protein expression (three independent ex vivo experiments). Yellow arrows mark representative nuclei of proliferative cells and blue arrows mark representative non-proliferative cells. Scale bar = 40 µm for histological images. Bars represent mean ± standard deviation (*n* ≥ 10 animals per group and *n* = 16 for Ki67 expression analysis). MH: magnetic hyperthermia; MNP; magnetic nanoparticle; 5FU: 5-fluorouracil; p.t.; post injection. Mann–Whitney U-Test and independent two-sample t-test indicated significant differences between the hyperthermia and chemotherapy combination (5FU-MNP/MH) group and the hyperthermia (MNP/MH) group with other groups (* *p* < 0.05, ** *p* < 0.01, and *** *p* < 0.001).

The measurement of tumor volume at specific points in time after local thermo-chemotherapeutic tumor treatment revealed that the combinatorial modality (5FU-MNP/MH) was considerably more effective than either therapies alone (MH or 5FU-MNP) (Figure 2a). The combinatorial treatment also resulted in complete tumor elimination in some cases (Figure 2b). In particular, there was a major contribution of the magnetic hyperthermia treatment component on the tumor volume. Namely, the magnetic hyperthermia therapy alone led to a strong reduction in tumor volume from day seven on after the first tumor therapy compared to the 5FU-MNP treatment and the untreated control group. Furthermore, there was no significant difference (*p* > 0.05) between the efficacy of 5FU-MNP treatment group and its sub-components (5FU and MNP in absence of magnetic hyperthermia) on tumor volume, and their respective outcomes were comparable to those of the untreated control group. In addition, there was a minor increase of tumor volume in the non-magnetic hyperthermia treated 5FU-MNP and the MNP exposed animal groups compared to the untreated control group at later post-therapy observation times (days 21 and 28 post first tumor therapy). Moreover, the cellular localization and the cytotoxic effect of 5FU-CS-MNP in human colon carcinoma cells (HT29) have been validated in an in vitro study. Namely, microscopic observations of the HT29 cells after incubation with 5FU-CS-MNPs (24 h, see methods) demonstrated the presence of MNPs attached to or internalized into the cells (Prussian blue staining of iron, Figure S1a). The cytotoxic effect of 5FU-CS-MNPs on the cells was reflected by a significant reduction of the cell viability (*p* < 0.001), whereas CS-MNP had almost no cytotoxic effect (Figure S1b).

Immunohistochemical staining revealed that the protein expression of the proliferation marker Ki67 was reduced when tumors were treated with the combinatorial therapy (5FU-MNP/MH) compared to the either therapies alone (5FU-MNP or MH). This significant reduction was observed on day 9 and 28 post first tumor therapy (*p* < 0.001 and *p* < 0.01 respectively). The Ki67 expression was not significantly different when comparing each therapeutic modality alone (*p* > 0.05, 5FU-MNP vs. MH) but slightly lower than that of the untreated control group. Additionally, the Ki67 protein expression in tumors after treatment with MNP or 5FU was comparable to those after treatment of tumors with 5FU coupled to MNP (5FU-MNP, Figure 2c). 

### 2.4. Tumor Cells Are Struggling for Survival as Result of Extensive DNA Damages Induced by the Combinatorial Thermo-Chemotherapeutic Tumor Treatment

The combinatorial thermo-chemotherapeutic tumor treatment led to a significantly elevated expression of both H2AX (used as indicator of DNA replication stress, *p* < 0.001 and *p* < 0.01, day 9 and day 28 post first tumor therapy, respectively) and the phosphorylated form of H2AX (phospho-H2AX, used as indicator of the presence of DNA double strand brakes, *p* < 0.05, day 9 post first tumor therapy) compared to the either therapies alone. In particular and in the view of either therapies alone, magnetic hyperthermia treatments caused a significantly increased H2AX protein expression (*p* < 0.05, day 9 post first tumor therapy) and only a slightly higher (but not significantly different) expression of phospho-H2AX compared to tumors of the animal group treated with 5FU-MNP (absence of hyperthermia). 

**Figure 3:**
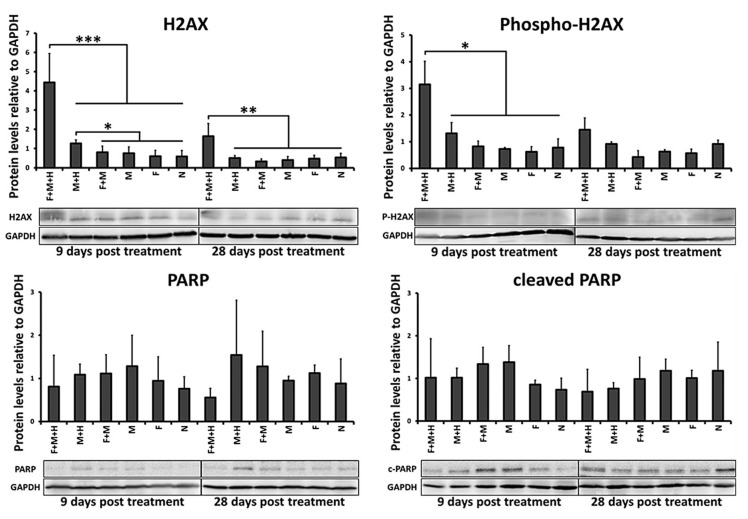
The combinatorial thermo-chemotherapeutic tumor treatment causes greater DNA damages than magnetic hyperthermia alone. Semi-quantitative analysis of protein expression using 3 to 5 independent ex vivo experiments. Representative images of protein bands after Western blot analysis of isolated HT29 xenograft tumor cells at 9 and 28 days after the first magnetic hyperthermia treatment (60 min at *H* = 15.4 kA/m, *f* = 410 kHz). GAPDH was used as loading control. F + M + H: combinatorial treatment group (5FU-MNP/MH); M + H: magnetic hyperthermia treatment alone group (MNP/MH); F + M: 5FU-MNP treatment group; M; MNP treatment group; F: 5FU treatment group; N: untreated group; GAPDH, glyceraldehyde 3-phosphate dehydrogenase; PARP, poly(ADP-ribose) polymerase; H2AX: H2A histone family member X; Independent two-sample t-test showed significant differences between the 5FU-MNP/MH and other groups (* *p* < 0.05, ** *p* < 0.01 and *** *p <* 0.001).

The expression of PARP and its cleaved form (c-PARP, used as indicator of the onset of apoptosis) did not change significantly (*p* > 0.05) in response to the combinatorial treatment compared to the either therapies alone. However, there was a tendency for lower long term PARP expression in tumors after treatment with the combinatorial treatment compared to either therapy alone (day 28 post first tumor therapy, Figure 3). Detail information about Figure 3 can be found in Figure S4.

**Figure 4:**
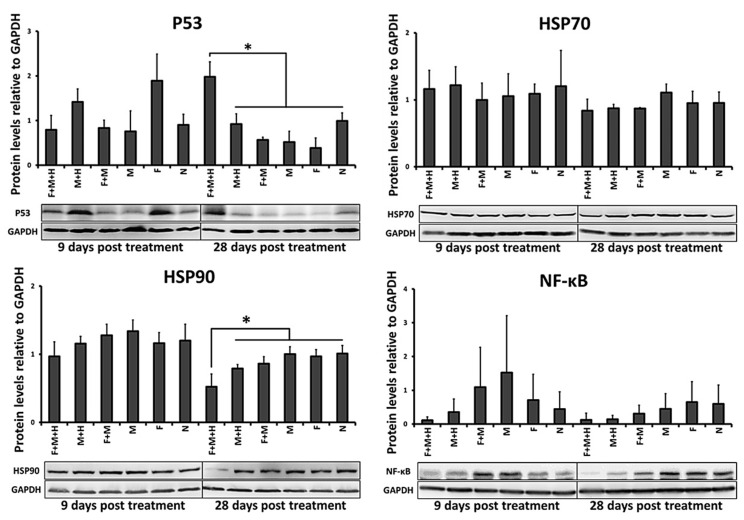
The surviving cells after the thermo-chemotherapeutic tumor treatment experience features of stress. Semi-quantitative analysis of protein expression using 3 independent ex vivo experiments. Representative images of protein bands after Western blot analysis of isolated HT29 xenograft tumor cells at 9 and 28 days after the first magnetic hyperthermia treatment (60 min at *H* = 15.4 kA/m, *f* = 410 kHz). GAPDH were used as loading control. F + M + H: combinatorial treatment group (5FU-MNP/MH); M + H: magnetic hyperthermia treatment alone group (MNP/MH); F + M: 5FU-MNP treatment group; M; MNP treatment group; F: 5FU treatment group; N: untreated group; GAPDH, glyceraldehyde 3-phosphate dehydrogenase; NF-κB: nuclear factor ‘kappa-light-chain-enhancer’ of activated B-cells; HSP: heat shock protein; independent two-sample t-test showed significant differences between the 5FU-MNP/MH and other groups (* *p* < 0.05).

Moreover, p53 protein expression (indicator of the onset of apoptosis and/or cell adaptive cell responses) was significantly increased (*p* < 0.05, day 28 after the first tumor therapy) in response to the combinatorial treatment (5FU-MNP and MH) compared to the either therapies alone. Regarding the either therapies alone, magnetic hyperthermia treatment per se resulted in a slightly (but not significantly) increased expression level of p53 compared to the group treated with 5FU-MNP at both points in time (9 and 28 days after the first tumor therapy, Figure 4).

In general, the expression levels of HSP70 and HSP90 (used as indicators for intracellular protein stabilization requirements after stress impulse) in tumors treated with the combinatorial treatment did not differ significantly (*p* > 0.05) from those in tumors treated with the either therapies alone. Only the HSP90 expression was significantly lower in tumors treated with the combinatorial treatment than in tumors treated with the either therapies alone (*p* < 0.05, day 28 post first tumor therapy; Figure 4).

The protein expression of NF-κB (used as an indicator of tumor cell proliferation and survival) following the combinatorial treatment was not significantly (*p >* 0.05) but by tendency reduced compared to its expression level in untreated tumors (9 and 28 days after the first tumor therapy, Figure 4). Shortly after the second tumor therapy on day 9, the expression of NF-κB in the combinatorial treatment group also tended to be lower than in the either therapies alone. Detail information about Figure 4 can be found in Figure S5. 

In addition, neither the combinatorial treatment group nor each of the either therapies alone showed a distinct impact on the expression of caspase-8, cleaved caspase-8 (c-caspase-8), and caspase-3 (indicators for the onset of intrinsic and extrinsic apoptotic pathways, Figure S2). Detail information about Figure S2 can be found in Figure S6.

Furthermore, the results showed no expression of the phosphorylated form of the receptor-interacting protein 3 (phospho-RIP3, used as an indicator of cell death via programmed necrosis (necroptosis)) neither in treated tumor cells nor in the untreated controls (Figure S3). Detail information about Figure S3 can be found in Figure S7.

### 2.5. The Combinatorial Thermo-Chemotherapeutic Tumor Treatment Impacts the Tumor Vasculature

**Figure 5:**
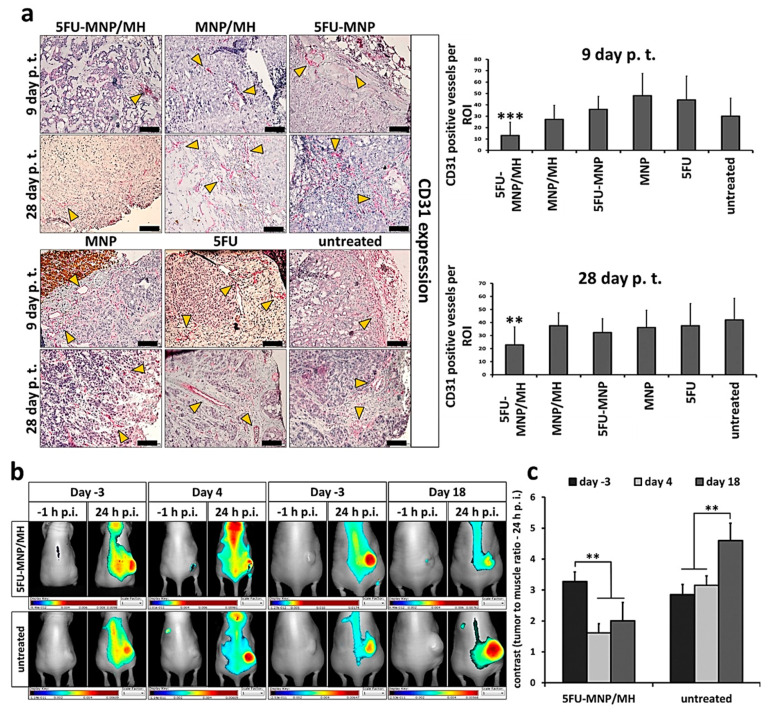
Tumor vascularization is impaired after treatment with the thermo-chemotherapeutic tumor treatment. (**a**) Reduced CD31 expression in tumors treated with the combinatorial therapy (5FU-MNP/MH) compared to the either therapies alone (5FU-MNP or MH). Depicted are representative histological micrographs of tumor tissue slices after immunohistochemical staining as well as semi-quantitative estimations of protein expression (*n* = 3 ex vivo experiments). Arrows marked representative CD31 positive vessels. (**b**) Representative light and NIR fluorescence images of tumor bearing animals after injection or not with 5FU-CS-MNPs (CS- and 5FU-CS-coated, 0.25 mg Fe per 100 mm^3^ tumor tissue and 0.22 to 0.55 mg 5FU per kg body weight) and exposure or not to AMF (60 min at *H* = 15.4 kA/m, *f* = 410 kHz). −1 h: before injection of the optical probe IRDye^®^ 800CW RGD targeting αvβ3 integrin, 24 h: time after injection of the optical probe. (**c**) Fluorescence contrast (tumor-muscle ratio) of the IRDye^®^ 800CW RGD optical probe at 24 h after injection, −3, 4, 18 days: time points with reference to the first tumor therapy. Images were obtained using the Maestro™ in vivo fluorescence imaging system. IRDye^®^ 800CW RGD injection concentration: 1 nmol per 25 g of mouse body weight; MH: magnetic hyperthermia; MNP; magnetic nanoparticle; 5FU: 5-fluorouracil; p.i.; post injection. Scale bar = 100 µm for histological micrographs. Bars represent the mean ± standard deviation of *n* = 28 for CD31 expression analysis and the mean ± standard error of *n* = 5 (days 4 and 18 post first tumor therapy), and *n* = 10 (day −3 post first tumor therapy). Independent two-sample t-test indicated significant differences between the groups (** *p* < 0.01 and *** *p* < 0.001).

The thermo-chemotherapeutic tumor treatment (5FU-MNP/MH) significantly decreased the protein expression of the endothelial cell marker CD31 (9 and 28 days after the first tumor therapy; *p* < 0.001 and *p* < 0.01, respectively) compared to either therapy alone and to its expression in the untreated tumor cells. Each of the therapeutic components alone (5FU-MNP or MH) were not as effective as the combinatorial treatment, whereas magnetic hyperthermia alone (MH) reduced the expression of the CD31 protein on day 9 post first tumor therapy to a somewhat higher extent compared to the 5FU-MNP treatment. Furthermore, the expression of CD31 was lower when tumors were treated with 5FU-MNP than when they were injected with MNP or 5FU alone (both in absence of hyperthermia, Figure 5a).

In tumors treated with the combinatorial treatment modality (5FU-MNP/MH), the in vivo whole-body NIRF-based detection of the endothelial proliferation marker αvβ3 integrin was significantly decreasing with ongoing time after therapy (days 4 and 18 post first tumor therapy compared to day −3 post first tumor therapy; *p* < 0.01). In contrast, in the untreated control group (therapy control group), the near infrared fluorescence-based detection of αvβ3 integrin was steadily increasing with ongoing time after therapy (days 4 and 18 compared to that on day −3 after the first tumor therapy; *p* < 0.01) (Figure 5b,c).

## 3. Discussion

In general, our data show that compared to the either therapies alone, the thermo-chemotherapeutic tumor treatment (5FU-CS-MNPs and magnetic hyperthermia) led to a substantial reduction of the tumor volume and decreased proliferation marker Ki67 in tumor cells. The strong cytotoxic activity of the coupled 5FU-CS-MNPs was corroborated in experiments with cultured HT29 tumor cells. Compared to the either therapies alone and to the untreated controls, the thermo-chemotherapeutic tumor treatment led to a substantial overexpression of H2AX and phospho-H2AX (indicators of DNA replication stress and DNA repair processes, respectively). The expressions of PARP and its cleaved form (c-PARP; indicator of the onset of apoptosis) were not altered when treating tumors with the thermo-chemotherapeutic modality. The late p53 protein expression (indicator of survival responses upon stress) was significantly increased, whereas the HSP70 and HSP90 protein expression (indicators of protein stabilization activities) did either not change or it was decreased, respectively, compared to either therapies alone. Furthermore, the expression levels of NF-κB (indicator of tumor cell proliferation, survival, and therapy resistance) was, by tendency, reduced due to the combinatorial treatment, while the expressions of caspase-8, c-caspase-8 and caspase-3 were not significantly affected in response to neither the combinatorial treatment or each of the either therapies alone. Moreover, tumors treated with the combinatorial treatment exhibited a distinctly reduced expression of the endothelial cell marker CD31 and endothelial cell proliferation marker αvβ3 integrin.

In this study, the increased size and decreased ζ-potential of the CS-MNPs after functionalization with 5FU (compared to non-functionalized ones) reflects the adsorption of 5FU molecules on the surface of the MNPs and prospectively a slight clustering of MNPs (changed ζ-potential). Moreover, the change of ζ-potential can influence the size of MNPs by inducing cluster formation to varying degrees [21]. In the view of the MNP exposure to an electromagnetic field, clustering can lead to a reduced Brownian relaxation [14]. Finally, functionalization of the CS- MNPs with 5FU had no significant effect on the iron contents and heating potential (SAR) of MNPs. This means that the MNPs exhibited favorable features for utilization in combined anti-tumor thermo-chemotherapeutic approaches.

We further observed that the thermo-chemotherapeutic tumor treatment “magnetic hyperthermia” and “5FU-MNP” (7.5 µg 5FU per 100 mm^3^ tumor and CEM43T90: 23.1 ± 10.8 min), compared to the either therapies alone (5FU-MNP-based chemotherapy group: 7.5 µg 5FU per 100 mm^3^ tumor, magnetic hyperthermia: CEM43T90: 19.7 ± 4.6 min) resulted in extensive tumor cell death, which in turn led to a substantial and persistent inhibition of tumor growth and reduced tumor volume. In some cases, a complete elimination of the tumor was seen, reflecting the profound therapeutic effect of the combinatorial treatment. Interestingly, the treatment of tumors with 5FU-MNP-based chemotherapy alone (absence of magnetic hyperthermia) had almost no effect on tumor volume. This finding can be attributed to the fact that the concentration of drug per mm^3^ tumor tissue was rather low (see above) to be effective as single therapy modality. On the other hand, hyperthermia has an additive effect on the toxicity of 5FU, since heat has been reported to potentiate the anti-tumorigenic action of drugs [6]. The most likely additive effect of hyperthermia on the cytotoxicity of 5FU is the disruption of DNA repair process, which is critical for cells suffering from 5FU-induced DNA double-strand breaks. Hyperthermia mainly interrupts with DNA repair by affecting its required enzymes [22] and 5FU exerts its cytotoxicity by inducing DNA damages [3]. The dose of intratumorally injected 5FU (attached to MNPs) in this study ranged from 0.22 to 0.55 mg/kg mouse body weight, which was dramatically lower than the dose of 5FU, which is commonly used in systemic chemotherapy (intravenous injection) for various types of cancer in mice (20–80 mg/kg mouse body weight) [23,24,25,26] and in humans (intravenous bolus injection, approximately 300–600 mg/m^2^ or 8–16 mg/kg body weight and intravenous infusion, approximately 600–3000 mg/m^2^ or 16–81 mg/kg body weight) [27,28,29,30,31]. This shows that our low dose of 5FU, when injected intravenously, will prospectively have no therapeutic effect.

Our in vitro examinations corroborated the fact that in spite of the low concentration of coupled 5FU (138 ± 14 µg 5FU per mg Fe), the 5FU-CS-MNPs were substantially cytotoxic to HT29 cells. This pronounced cytotoxicity of the 5FU-CS-MNPs can be related to the dipole-dipole inter-molecular interactions (hydrogen bonding), namely interactions between hydrogen atoms of chitosan hydroxyl-groups and of oxygen in the 5FU molecules, which are sensitive to the surrounding environment and temperature. A study showed that almost 100% of 5FU coupled to chitosan coated MNPs via hydrogen bonding are released within 6 h when kept at a pH of 6.8 and a temperature of 37 °C [32]. In consequence, we assume that upon addition of 5FU-CS-MNP to the cell culture medium in in vitro experiments (pH = 7.4), 5FU molecules were released from the MNPs into the culture medium and that they entered the cells via the same mechanism as uracil is able to do [3]. Once 5FU-CS-MNPs are internalized in cells (our microscopic analyses showing MNPs attached to and/or internalized into HT29 cells), the distinct reduction of pH in the endo-lysosomes (pH ≈ 3–5.5) will release the remaining 5FU attached to the MNPs. A similar situation applies to the in vivo situation in tumors upon intratumoral application, since the extracellular matrix of tumor tissues is known to exhibit a pH of around 6. An acidic environment favors the release of bound 5FU from CS-MNPs [33], because firstly, a pH change leads to swelling and shrinking of the chitosan molecules [17], and secondly the hydrogen bonding between chitosan coating of MNPs and 5FU molecules weaken up at medium acidic environments, and it results in an facilitated release of the drug from the polymer. We expect that such a facilitated the release of 5FU from the MNPs accounted for the massive tumor cell death beyond the impact of (magnetic) hyperthermia as observed by the reduced tumor volumes in vivo and the high tumor cell cytotoxicity in vitro. We conclude that most 5FU molecules are released from CS-MNPs prior to the hyperthermia application either to the tumor extracellular matrix or within the endo-lysosomes, which means that 5FU first performs its anticancer function and the action of hyperthermia follows it. In agreement with the mentioned observations, colon adenocarcinoma cells, which were able to survive (or still struggling for survival) the therapeutic impact, exhibited a decreased proliferation activity, since the proliferation marker Ki67 was considerably lower in tumors treated with the combinatorial treatment than those treated with the either therapies alone. This implies that the proposed combinatorial treatment has distinct feasibilities to impair the colon cancer proliferative potential.

We further demonstrated that the expression of H2AX was significantly higher in tumors treated with the thermo-chemotherapeutic treatment than in those treated with either therapies alone or those not treated at all (untreated control tumors), both at early and late post-observation times (day 9 and day 28 after the first tumor therapy, *p* < 0.001 and *p* < 0.01, respectively). The increased presence of H2AX in its non-phosphorylated (and non-chromatin-bound) state was considered as indicator of DNA replication stress. Namely, its overexpression is a consequence of DNA replication interferences and DNA damages, and these processes are reported to be associated with an increased production of reactive oxygen species (ROS) and thus related to a higher tendency of cells to die [34,35,36]. In fact, DNA damage induces the production of ROS at high levels [37]. Excess of ROS in turn, can further damage DNA, destroy proteins and induce membrane peroxidation, both of which ultimately lead to cell death [38]. Moreover, we have previously reported a significant two- to three-fold increase of ROS after magnetic hyperthermia treatments of a pancreatic cancer cell line [39]. On the other hand, it has been reported that 5FU stimulates the production of ROS on its own [40]. The excess of ROS (e.g., due to hyperthermia, 5FU treatment and in response to the overexpression of H2AX) is very well able to induce cell death through ROS-dependent mechanisms, e.g., via ROS-triggered activation of KEAP1-PGAM5-AIFM1 pathways (oxeiptosis, [41]). Therefore, we conclude that the therapeutically induces ROS production in tumor cells plays a crucial role in the regulation of cell death after tumor therapy. 

In addition, the expression of phosphorylated H2AX was significantly higher (*p* < 0.05) after thermo-chemotherapeutic treatment compared to either therapy alone and the untreated control tumors on day 9 after the first tumor therapy. A rapid and intense phosphorylation of H2AX, a key step in DNA damage response mediated by Ataxia-telangiectasia mutated kinase (ATM), ataxia telangiectasia and Rad3-related protein (ATR) and DNA-dependent protein kinase, catalytic subunit, (DNA-PK), is generally considered to be an indicator of the presence of DNA double-strand breaks [35,42]. In this study, the overexpression of phosphorylated H2AX clearly indicates that severe DNA damages (in particular by DNA double-strand breaks) and consequently distinct DNA repair disturbances were induced. Such effects are the particular consequence of the exposure of tumor cells to hyperthermia, which is known to cause DNA damage and indirectly by producing ROS, which also interferes with DNA replication (e.g., by utilization of hyperthermia at temperatures above 43 °C for 30 min [22]). On the other hand, the presence of 5FU, which was released from the CS-MNPs after intratumoral injection, also induces DNA double-strand breaks and interferes with DNA replication process [3,43]. A relatively high expression of phosphorylated H2AX after the combinatorial treatment compared to the either therapies alone and the untreated control tumors indicates that the surviving cells are still facing a certain degree of DNA damage or DNA repair failure, suggesting that tumor cells are probably more sensitive to further stress impacts. In general, compared to the either therapies alone, the combinatorial treatment seems to be very effective in inducing DNA damage and disrupting the DNA replication process and consequently in causing cell death, as we did not observe comparable outcomes with either magnetic hyperthermia or 5FU-MNP-based chemotherapy alone.

Rather unexpectedly, the expression of PARP and c-PARP did not change significantly (*p* > 0.05) after treatment of tumors with the chemo-therapeutic treatment compared to the either therapies or to their normal expression levels (untreated control tumors). Full length PARP is usually involved in DNA repair and is a key protein in phosphorylation of H2AX via activating ATM kinase [44,45]. It is expected that PARP is degraded after having fulfilled its role. This could explain the reduced PARP expression at the long post-therapy observation time. On the other hand, cleavage of PARP, which is mainly done by caspase-3, indicates that cells are undergoing apoptosis [46]. In this study, the unchanged expression of c-PARP after the combinatorial treatment and the either therapies alone, compared to its normal expression in untreated control tumors suggested a low probability of apoptosis as the main known mechanism of cell death. In agreement with this argumentation line, we showed that the expressions of caspase-3, caspase-8 and c-caspase-8, which are known markers of intrinsic and extrinsic apoptosis pathways [47], were unchanged or relatively decreased due to combinatorial tumor treatment compared to the either therapies alone and to the untreated control tumors. These results confirm, in accordance with the low expression of c-PARP, that the classical caspase-dependent apoptosis pathway is not the main mechanism of death in tumors treated with the combinatorial treatment. Additionally, since no phosphorylated RIP3 could be detected, there are no hints to cell death via necroptosis [48]. More probable are ROS-dependent—but caspase-independent—pathways of cell death, like oxeiptosis [41] in tumor areas surrounding MNPs as reported above. 

The protein expression of p53 was distinctly increased in tumors treated with the thermo-chemotherapeutic treatment and it was clearly higher than in tumors treated with the either therapies alone. This observation was particularly true for later periods in time after treatment. The HT29 colon carcinoma cell line, as seen in up to 50% of human cancers cells, is known to express a mutated p53 protein [49]. Mutant p53 usually contributes in cancer cell survival by regulating the adaptive response to stress conditions [50]. In this study, the significantly increased level of p53 (28 days post first tumor therapy) in tumor cells treated with the combinatorial treatment seems to be a reaction against the therapeutically-based stress stimuli. The extensive DNA damages and DNA repair disturbances (see above) induced by the combination of magnetic hyperthermia and 5FU-MNP-based chemotherapy could well have been the reason for the overexpression of p53 in surviving cells. In this context, we hypothesize that overexpression of mutated p53 “helps” tumor cells to overcome different stress conditions. This is in agreement with the almost unchanged PARP expression in treated tumors. Namely in presence of high levels of DNA damage, mutant p53 inactivates the stress-sensor kinase ATM and shifts the DNA repair process more towards a PARP-dependent pathway, in particular by enhancing the stress-related function of PARP [44,50]. Inactivation of ATM kinase by p53 explains the reduced expression of phosphorylated H2AX in surviving cells with increasing time after the combinatorial treatment, since ATM mediates the phosphorylation of H2AX [35]. Summarizing, the overexpression of p53 observed at later periods of time after the combinatorial tumor treatment can be interpreted as a protective response of cells which have survived extensive DNA damage. This implies that, compared to the either therapies alone, the combinatorial treatment was more effective in inducing long-lasting cellular stress responses in surviving tumor cells. In the view of a prospective therapy in humans, repeated therapy sessions (more than two times) will be necessary in order to completely inactivate the tumor cell feasibility to recover from the single stress impacts. 

Interestingly, there was a rather low impact of the thermo-chemotherapeutic tumor treatment on the HSP (HSP70, HSP90) protein expression levels. The significantly reduced expression of the HSP90 (*p* < 0.05, day 28 post first tumor therapy) could be related to a high protein “consumption” of those tumor cells which were struggling for survival. It is of note that HSP70 and HSP90 are inducible molecular chaperons [51]. They exert a chaperoning role by refolding or targeting proteins for degradation [52], which have previously been damaged as a consequence of heat. Having fulfilled their roles, proteins are usually degraded. In the long term, a reduced chaperoning of HSPs’ target proteins, which are particularly responsible for DNA repair, protein homeostasis, chromatin remodeling, etc., can very well lead to destabilization of tumor cells, as well as to an increased sensitivity of those cells to further anti-cancer treatments. This is in analogy to the expected effects of HSP inhibitors in cancer therapy [52]. Therefore, we conclude that tumor cells surviving the combinatorial treatment are more sensitive to further stress conditions, since the expression of HSP is remarkably decreased (HSP90) or it did not change (HSP70). 

The expression of NF-κB (used as an indicator of tumor cells proliferation, survival and therapy resistance [53]) was persistently reduced, at least by tendency, in response to the thermo-chemotherapeutic treatment, compared to the either therapies alone and to its expression in the untreated tumors with increasing time after therapy. This persistently reduced NF-κB expression, although only by tendency, can be accounted for a long-lasting reaction of cells against stress. Moreover, the reduced NF-κB expression caused by the combinatorial treatment could well reflect an increased sensitization of surviving cells to the action of 5FU, as it is already reported that NF-κB inhibition helps to overcome resistance of colon cancer cells to 5FU [54]. These results highlight a further therapeutic effect of the combinatorial treatment and suggest that one or a few additional therapy sessions with 5FU (systemic or local administration) could lead the resistant cells to death.

We finally demonstrated that the thermo-chemotherapeutic treatment caused a persistent reduction of endothelial cell marker CD31 protein expression with increasing time after therapy compared to the either therapies alone. CD31 is a marker for endothelial cells and reflects the development and distribution of vessels in different parts of the body as well as in tumors [55]. Moreover, the combinatorial tumor treatment caused a sustained reduction of the proliferation marker αvβ3 integrin in tumor endothelial cells after therapy. In contrast, presence of αvβ3 integrin in the untreated tumors was steadily increasing. αvβ3 integrin is a transmembrane receptor, which is overexpressed on tumor endothelial cells and was reported to be associated with angiogenesis in cancer [56]. This means that the combinatorial treatment was able to damage the tumor vascular system, which could be due to a synergistic effect of the combination of heat and 5FU; this is another therapeutic effect that surpasses the combinatorial treatment over the either therapies alone. It is reported that 5FU, not only kills tumor cells, but is also toxic to endothelial cells and inhibits VEGF-induced angiogenesis in tumor [57,58]. An impairment of the tumor vascular system is beneficial for anti-tumor therapies, since it leads to a restricted supply of nutrients and oxygen due to reduced blood flow. Beyond the influence of thermo-chemotherapy on tumor vasculature, heat could affect the components of the extracellular matrix as well, particularly the collagen fibers [59]. Our group recently reported that mild iron oxide MNP-based hyperthermia (40 °C, 42 °C, 60 min) considerably reduces the integrity of collagen fibers in heterotumor spheroids composed of pancreatic cancer cells and fibroblasts compared to control collagen fibers held at 37 °C [60]. Other studies have shown the same effect of temperature rise on collagen architecture in extracellular matrix of epidermoid carcinoma tumors in vivo [61,62]. These findings imply that if there is some degree of desmoplasia in our heterotopic colon tumors, heat may damage the collagen fibers in their extracellular matrix. However, more detailed understanding of the impact of combination of local hyperthermia and chemotherapy on extracellular matrix and collagen fibers requires comprehensive investigations.

## 4. Materials and Methods

### 4.1. Magnetic Nanoparticle, Functionalization with 5-Fluorouracil and Characterization

Superparamagnetic iron oxide nanoparticles (MNP) were coated with chitosan. 5-fluorouracil (5FU) powder for coupling to chitosan-coated MNP (named as CS-MNP) was purchased from Sigma-Aldrich (Sigma-Aldrich Chemie GmbH, Steinheim, Germany). To functionalize the surface of CS-MNPs with 5FU, 500 µL of 5FU diluted in 1 N NH_4_OH solution at a concentration of 10 mg/mL was added to 500 µL MNP suspension (1 mg Fe/mL). The MNP suspension was gently mixed on a shaker (Heidolph polymax 2040, Heidolph Instruments GmbH & CO. KG, Schwabach, Germany) for 20 min at room temperature and then the MNPs were separated magnetically from the solution and re-suspended in distilled water. The pH of CS-MNPs before and after functionalization with 5FU was measured using pH color-fixed indicator strips from MACHEREY-NAGEL GmbH & Co. KG, Düren, Germany. The functionalized MNPs were named as 5FU-CS-MNP. The concentration of the coupled 5FU was measured via a UV-vis spectrometer (Ultrospec 4300 pro UV/Visible Spectrophotometer) by detecting 5FU absorption at 265 nm wavelength. For intratumoral injection of free 5FU, a 5FU injection solution (Accord Healthcare GmbH, Munich, Germany) was used. All procedures regarding the physicochemical characterization of 5FU-CS-MNPs and CS-MNPs as well as the measurement of iron content through atomic absorption spectroscopy (AAS) are described elsewhere [21]. Briefly, dynamic light scattering (DLS) method was used to measure the size, ζ-potential, and polydispersity index (PdI) of 5FU-CS-MNPs and CS-MNPs (dispensed in water) using the Zetasizer Nano ZS (Malvern Instruments GmbH, Herrenberg, Germany). To quantify the iron content of through AAS, 32% HCL solution (*v*/*v*, Carl Roth GmbH, Karlsruhe, Germany) was added to MNPs and incubated for 30 min at room temperature. Then, 10% trichloroacetic acid (*w*/*v*, Carl Roth GmbH, Germany) was added and was centrifuged at 3720× *g* for 5 min. Finally, the supernatant was taken to measure the iron content of MNPs using an AAS 5 FL spectrometer (Analytik Jena AG, Jena, Germany). 

### 4.2. Heating Potential of MNPs

To calculate the specific absorption rate (SAR), the temperature increase per unit of mass of 5FU-CS-MNPs and CS-MNPs dispersion (200 µL) after exposure to an alternating magnetic field (AMF) (AMF specifications: *H* = 15.4 kA × m^−1^, *f* = 435 kHz) was measured using optical fiber temperature sensors (TS5 & FOTEMPMK-19, Optocon AG, Dresden, Germany). The following equation [63] was used for calculations:

(1) $$\mathrm{SAR}=c*\frac{m_{F}}{m_{P}}*\frac{\Delta T}{\Delta t},$$

in which *c* equals the MNP dispersion specific heat capacity, *m_F_* represents the fluid mass, *m_P_* is the mass of MNP, and Δ*T*/Δ*t* corresponds to the highest linear slope value at initial times upon switching on the AMF.

### 4.3. Cell Culture

HT29 cell line (human colorectal adenocarcinoma from DSMZ GmbH, Braunschweig, Germany) was cultured at 37 °C with 5% (*v*/*v*) CO_2_ and 95% (*v*/*v*) humidity, in DMEM/Ham’s F12 (1:2), purchased from Gibco (Life Technologies, New York, NY, USA) with 10% (*v*/*v*) FBS (Thermo Fisher Scientific Inc., Waltham, MA, USA).

### 4.4. Cell Viability Determination, Prussian Blue Staining of Iiron and Microscopy

To corroborate the cytotoxicity of 5FU-CS-MNP, we measured the viability of HT29 cells after incubation with of 5FU-CS-MNP (100 µg Fe and 13.8 µg 5FU per mL fluid, 24 h incubation time) using alamarBlue^®^ assay. To validate the cell internalization and localization of MNPs in cells, Prussian blue staining method was implemented, and images were acquired at 60× magnification with an EVOS xl AMG microscope (PEQLAB, Erlangen, Germany). The methods used for determination of cell viability, Prussian blue staining and microscopy were described elsewhere [21].

### 4.5. Animals and Ethics

In total 8 weeks old female athymic nude mice (Hsd: Athymic Nude-Foxn1^nu^) from Envigo (Envigo RMS GmbH, Venray, The Netherlands) were used in this study. All experiments were approved by the regional animal care committee (Reg.-Nr. 02-058/16; Thüringer Landesamt für Verbraucherschutz, Bad Langensalza, Germany) and carried out in accordance with international guidelines for the ethical use of animals. Isoflurane CP (CP-Pharma, Burgdorf, Germany) at 2% (*v*/*v*) concentration was used to anaesthetize the animals during the experimental interventions. Tumor induction was done by subcutaneous injection of 2 million HT29 cells suspended in 100 to 120 µL Matrigel™ (Becton, Dickinson and Company, Franklin Lakes, NJ, USA). Once the tumor volume had reached a volume of 100 to 250 mm^3^, the experiments were started. Tumor volume was measured by utilization of the formula *V = π*/*6 × (length × width × height of the tumor)* throughout all experiments [64].

### 4.6. Animal Groups, General In Vivo Experimental Procedure

**Figure 6:**
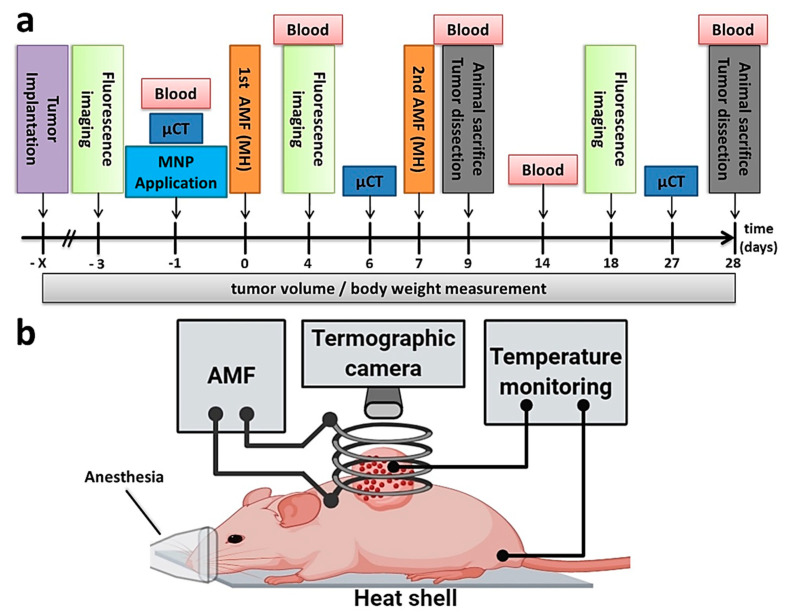
Procedure for in vivo experiments and experimental setup for magnetic hyperthermia. (**a**) Schematic illustration of the in vivo experiments conducted. (**b**) Magnetic hyperthermia was performed by exposure of intratumorally injected MNPs (red dots) (0.25 mg Fe/100 mm^3^ tumor and 0.22 to 0.55 mg 5 FU/kg body weight) to AMF (*H* = 15.4 kA/m, *f* = 410 kHz). AMF: alternating magnetic field; MH: magnetic hyperthermia; µCT: micro computed tomography; MNP: magnetic nanoparticle. Figure 6b is created with BioRender.com.

In this study, six independent animal groups were defined as follows: group (1) (5FU-MNP/MH): the combinatorial treatment group (with 5FU-CS-MNP, exposed to AMF, *n* = 11), group (2) (MNP/MH): the magnetic hyperthermia treatment alone group (with CS-MNP, exposed to AMF, *n* = 11) to monitor the contribution of magnetic hyperthermia in the combinatorial treatment, group (3) (5FU-MNP): the 5FU-CS-MNP-based chemotherapy alone group (with 5FU-CS-MNP, without AMF, *n* = 10) to study the impact of 5FU when coupled with CS-MNP in the combinatorial treatment, group (4) (MNP): the CS-MNP injected group (with CS-MNP, without AMF, *n* = 11) to study the effects of CS-MNP alone as part of the 5FU-CS-MNP treatment, group (5) (5FU): the 5FU injected group (with free 5FU, without AMF, *n* = 11) to monitor the contribution of 5FU as part of the 5FU-CS-MNP treatment, and group (6) (untreated): the untreated group (no MNP, no 5FU, no AMF, *n* = 11) to monitor the overall therapeutic outcome of the treatments. Intratumoral injections (0.25 mg Fe per 100 mm^3^ tumor tissue and 0.22 to 0.55 mg 5FU per kg body weight) were done on day −1 post first tumor therapy (one day before the first tumor therapy) (Figure 6a). To monitor the effects of treatments on the animals’ health, blood samples were taken on days −1, 14, and 28 after the first tumor therapy. Tumor volume and body weight of the animals were measured every 3 to 4 days throughout the post-treatment observation time. The tumor volume on day −3 after the first tumor therapy was considered as reference for the changes of tumor volume during the post-treatment observation period, and data were normalized to this reference value (relative tumor volumes). Animals were sacrificed on day 9 and day 28 post first tumor therapy (early and late post- observation periods, respectively). Magnetic hyperthermia was performed on day 0 and 7 post first tumor therapy by exposure of tumors to an AMF (*H* = 15.4 kA/m, *f* = 435 kHz) for 60 min. During the magnetic hyperthermia treatment, the body temperature (rectal) of the animals and the temperature of the tumor surface were monitored by utilization of optical fiber temperature sensors as well as an infrared thermographic camera (NEC Avio Infrared Technologies Co. Ltd., Tokyo, Japan) (Figure 6b).

### 4.7. Temperature Dose

The estimation of the temperature dose applied to the tumors during the magnetic hyperthermia treatment was done by utilization of the tumor surface temperatures from the thermal images (heat maps) taken with the thermographic camera during magnetic hyperthermia. Using heat maps acquired during the hyperthermia treatment, a ROI was plotted around each tumor to extract the temperature data per pixel. Then, in accordance with Sapareto and Dewey 1984 [10], T90 temperatures and cumulative equivalent minutes at 43 °C (CEM43T90) were estimated. These calculations were undertaken under the assumption that the heating map at the distal tumor surface was representative for the whole tumor. 

### 4.8. Micro Computed Tomography In Vivo Imaging of Intratumoral MNP Distribution

The intratumoral MNP distribution of each animal during in vivo hyperthermia treatment was analyzed by performing micro computed tomography (μCT) imaging (TomoScope Synergy Twin, CT Imaging, Erlangen, Germany) immediately after injection of MNP as well as, on days 7 and 28 after the first tumor therapy. µCT imaging was performed using a low dose radiation protocol (29 s, 65 kV). The distribution of MNP was analyzed with the Imalytics Research software (Philips Technologie, Aachen, Germany).

### 4.9. In Vivo Imaging of Tumor αvβ3 Integrin

For whole body near infrared fluorescence imaging, anesthetized mice were intravenously injected with 1 nmol per 25 g body weight of the commercially available contrast agent IRDye^®^ 800CW RGD Optical Probe (LI-COR^®^ Biosciences, Lincoln, NE, USA). This was done to target αvβ3 integrin on endothelial tumor cells on days −1, 4 and 18 post first tumor therapy. Image acquisition was carried out via Maestro^TM^ in vivo fluorescence imaging system (Cri-InTAS, Woburn, MA, USA) at 24 h after the injection of optical probe (excitation filter: 671–705 nm; emission filter: 750 nm long-pass). Fluorescence intensities of tumors relative to background (muscle) were obtained by selecting regions of interest (ROIs, 303 to 906 pixels) on tumors or upper leg regions of mice. For each of the ROIs, the fluorescence intensities were calculated as an average signal (scaled counts/s), which makes the analysis comparable.

### 4.10. Analysis of Protein Expression of Tumor Tissue

#### 4.10.1. Immunohistochemistry of Ki67 and CD31 in HT29 Tumors

Methanol-stabilized 5% (*v*/*v*) formaldehyde solution (Otto-Fischer GmbH, Saarbrücken, Germany) was used for fixation of HT29 tumors. Afterwards, tumors were embedded in paraffin, sliced into three-micron thick sections using microtome system (Microm HM 340E, Thermo-Fischer) and placed on (poly-l-lysin)-coated glass slides. Sliced sections were blocked with avidin and biotin. Blocked sections were incubated with primary anti-human Ki67 or primary anti-mouse CD31 antibody (1:500, both from Abcam, Berlin, Germany), rinsed in Tris-Buffered Saline added with 0.1% (*w*/*v*) Tween 20 (TBST), and later on incubated with a secondary goat anti-rabbit IgG (H + L) biotin antibody (1:2250, Dianova GmbH, Hamburg, Germany). Streptavidin Alkaline Phosphatase (Biozol, Eching, Germany) and a Chromogen (Dako, Glostrup, Germany) were used for antigen detection. Sections were counterstained with hematoxylin (Sigma-Aldrich, Karlsruhe, Germany). For semi-quantitative analysis, the CellSens Dimension software from Olympus (Olympus GmbH) was used. Immunohistochemical staining was performed for tumor tissue of two mice per experimental group. Two independently stained slides per mouse were imaged to quantify the expression of Ki67 (20× magnification) by setting four and seven ROIs per slide. For CD31 as marker for tumor vascularity was estimated through the hot spot analysis by Chalkley count [65].

#### 4.10.2. Extraction of Tumor Proteins and Protein Expressions Analyses

Extracted tumors from sacrificed animals were washed on ice-cooled phosphate buffered saline (PBS) and weighted. Afterwards, equal weight amount of tumors of each particular animal group were pooled (*n* ≥ 4 for animals sacrificed on day 9 post first tumor therapy and *n* ≥ 5 for animals sacrificed on day 28 post first tumor therapy) and homogenized in RIPA lysis buffer containing protease inhibitor and phosphatase Inhibitor (both from Roche Diagnostics GmbH, Mannheim, Germany) using gentleMACS™ Octo Dissociator (Miltenyi Biotec B.V. & Co. KG, Bergisch Gladbach, Germany). After centrifugation at 15,680× *g* for 5 min, the supernatant was taken for determination of protein concentration via Bradford assay. The absorption of the supernatant was measured at 595 nm using a plate reader (Tecan Infinite M1000 Pro, Tecan Group Ltd., Männedorf, Switzerland). Sodium dodecyl sulfate polyacrylamide gel electrophoresis (SDS-PAGE, 12% or 15% (*w*/*v*) SDS gels) and Western blotting (Immobilon^®^-P, Merck Millipore Ltd., Carrigtwohill, Ireland) were performed to separate and detect proteins in the samples. 1% (*w*/*v*) bovine serum albumin (Albumin Fraction V, Carl Roth GmbH) dissolved in PBS and added with 0.1% (*w*/*v*) Tween-20 (PBST) was used to block the blotted membranes. The blocked membranes were incubated with primary rabbit antibodies against H2AX, phosphorylated H2AX (Ser139; phospho-H2AX), PARP, cleaved PARP (c-PARP), HSP90, NF-κB, p53, caspase-8, cleaved caspase-8 (c-caspase-8), caspase-3, and phosphorylated RIP3 (Ser227; phospho-RIP3) (all 1:1000, Cell Signaling Technology Inc, Danvers, MA, USA) and HSP70 (1:1000, Dianova GmbH, Hamburg, Germany) overnight at +4 °C. GAPDH used as a loading control protein; it was detected using primary anti-GAPDH antibodies (1:1000, from mouse or rabbit, Santa Cruz Biotechnology Inc., Dallas, TX, USA). In the next step, the membranes were incubated with secondary IgG-HRP antibody (goat anti-mouse 1:5000, Santa Cruz Biotechnology Inc. or anti-rabbit 1:10,000, Dianova GmbH, 1 h at room temperature). Finally, protein band detection was performed with enhanced chemiluminescence (EMD Millipore Corporation, Burlington, MA, USA and ImageQuant™ LAS 4000, Cytiva Europe GmbH, Freiburg, Germany). Semi-quantitative analysis of protein expression was done using ImageJ software. Purified proteins from human melanoma cell lines MDA-MB-435s (CLS Cell Lines Service GmbH, Eppelheim, Germany) was used as positive control for expression of phospho-RIP3.

### 4.11. Statistics

Data are presented as mean ± standard deviation or error of the mean. Statistical significance between the experimental groups was determined by utilization of an independent two-sample t-test or Mann-Whitney U-Test using IBM^®^ SPSS^®^ Statistics 21 software. *p*-values less than 0.05 were considered to be statistically significant.

## 5. Conclusions

In this study, we demonstrated that the treatment of colorectal cancer with the thermo-chemotherapeutic treatment composed of magnetic hyperthermia and 5FU-MNP-based chemotherapy was much more effective and led to a better therapeutic outcome in tumor bearing mice than magnetic hyperthermia or 5FU-MNP-based chemotherapy alone, as reflected by the distinctly reduced tumor volume and inhibited tumor cell proliferation (i.e., Ki67 protein expression). We could further show that the combinatorial treatment induced extensive DNA damages and disrupted of DNA repair processes in heterotopic colon tumors, which led to extensive cell death shortly after the second tumor therapy. The unchanged or only slightly decreased expression of c-PARP, caspase-3, caspase-8 and c-caspase-8 indicate that the classical caspase-dependent apoptosis was not the leading pathway of cell death after the combined thermo-chemotherapeutic treatment. No sign for necroptosis could be detected as well. We hypothesize that the presence of ROS plays distinct role in post-therapeutic cell responses, which prospectively leads, at least in parts, to cell death via oxeiptosis. Additionally, tumor cells surviving the thermo-chemotherapeutic treatment showed several stress responses, such as late overexpression of p53. Moreover, the decreased or unaltered expression of HSP70 and HSP90, PARP, and NF-κB prospectively renders treated tumor cells more sensitive to further therapy-based stress conditions. The thermo-chemotherapeutic treatment finally led to a distinct impairment of tumor vasculature. For these reasons, a very effective elimination of colon cancers seems to be feasible by utilization of repeated thermo-chemotherapeutic therapy sessions in the long-term.

## Figures and Tables

**Table 1:** Main features of the MNPs used in the present study. CS: Chitosan; 5FU: 5-fluorouracil; MNP: magnetic nanoparticle; PdI: polydispersity index; DLS: dynamic light scattering; AAS: atomic absorption spectrometry; SAR: specific absorption rate. The numbers are reported as mean ± standard deviation, *n* = 3.

Parameters	CS-MNP	5FU-CS-MNP
Size/Intensity (nm)	98 ± 2	176 ± 7
Size/Number (nm)	52 ± 2	92 ± 9
PdI	0.14 ± 0.01	0.17 ± 0.01
ζ-potential (mV)	20.1 ± 3.8	−27.8 ± 0.5
Iron content (mg Fe/mL)	1.2 ± 0.1	1.1 ± 0.2
SAR (W/g Fe)	515 ± 34	462 ± 70

## Supplementary Materials

The following are available online at https://www.mdpi.com/2072-6694/12/9/2562/s1, Figure S1: 5FU-CS-MNPs internalized and/or attached to tumor cells and inhibited tumor cell growth, Figure S2: Expression of caspases, involve in classical caspase-dependent apoptosis, Figure S3: Thermo-chemotherapeutic colon cancer treatment does not trigger necroptosis, Figures S4–S7: Detail information about Figure 3 and Figure 4, Figures S2 and S3.
