# Supplementary material for: The Treatment of Heterotopic Human Colon Xenograft Tumors in Mice with 5-Fluorouracil Attached to Magnetic Nanoparticles in Combination with Magnetic Hyperthermia Is More Efficient than Either Therapy Alone
Source: Cancers (Basel). 2020 Sep 9;12(9):2562. doi: 10.3390/cancers12092562 (PMC7566013; doi:10.3390/cancers12092562)
Supplement: Supplementary file 1 [file cancers-12-02562-s001.pdf]

Supplementary Materials

The Treatment of Heterotopic Human Colon Xenograft Tumors in Mice With 5-Fluorouracil Attached to Magnetic Nanoparticles in Combination with Magnetic Hyperthermia Is More Efficient Than Either Therapy Alone

Mohammad Dabaghi, Rainer Quaas and Ingrid Hilger

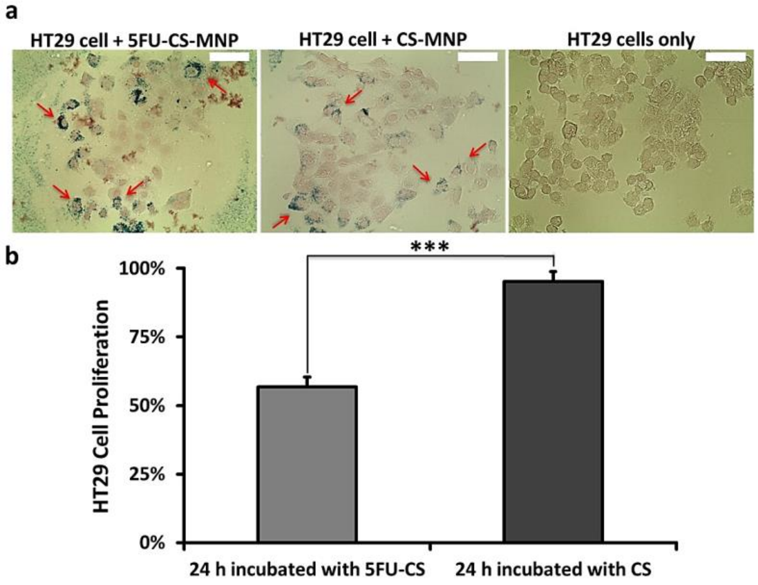

Figure S1. 5FU coupled to chitosan coated MNPs (5FU-CS-MNP) accumulated in human colon adenocarcinoma and inhibited their cell growth. (**a**) Light microscopic images after Prussian blue staining of iron after exposure of HT29 cells incubated or not with 5FU-CS-MNP and CS-MNPs (100 µg Fe per mL), (**b**) The viability of HT29 cells (measured by the alamarBlue® assay) after 24 h of incubation with 5FU-CS-MNPs or CS-MNPs. Arrow: Prussian blue staining of iron, CS: Chitosan; 5FU: 5-fluorouracil; MNP: magnetic nanoparticle; FSB: fetal bovine serum. Scale bar: 50 µm, $n = 5$, bars represent mean ± standard deviation. Independent two-sample t-test indicated significant differences between the groups with *** $p < 0.001$.

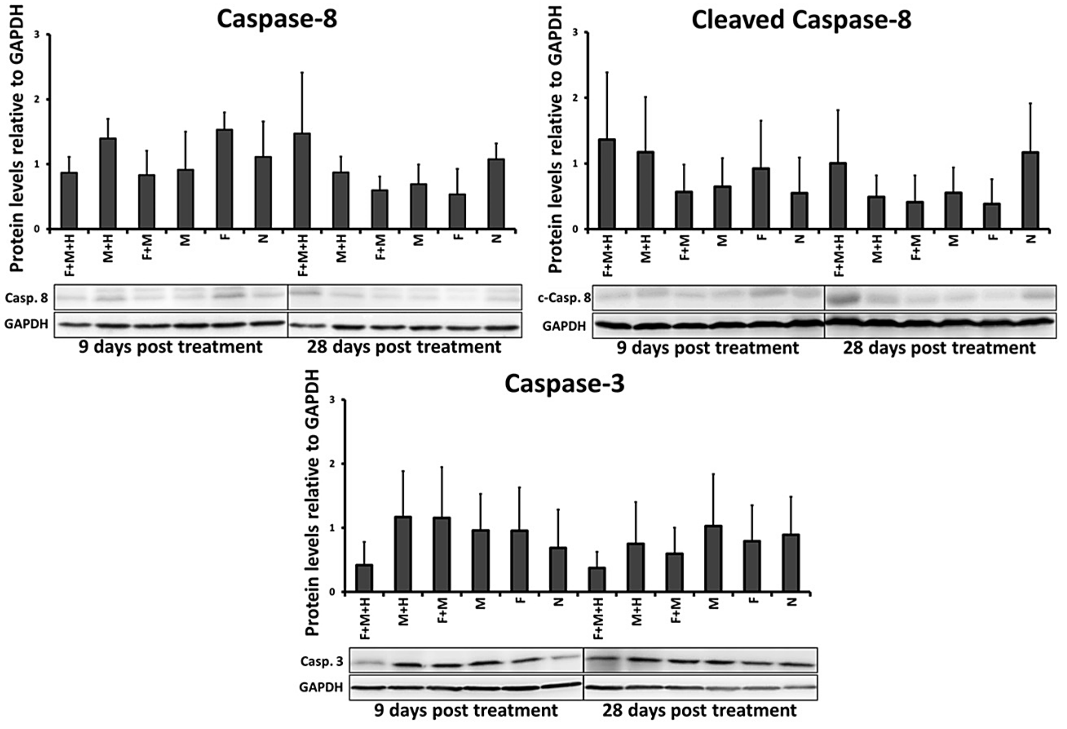

Figure S2. The combined thermo-chemotherapeutic therapy does not distinctly impact the expression of caspases involved in classical caspase-dependent apoptosis. Semi-quantitative analysis of protein expression of 3 independent ex vivo experiments. Representative images (of at least 3 independent experiments) of protein bands after Western blot analysis of HT29 xenograft tumor cells isolated at 9 and 28 days after the first magnetic hyperthermia treatment (60 minutes at H = 15.4 kA/m, f = 410 kHz). GAPDH were used as loading control. F + M + H: combinatorial treatment group (5FU-MNP/MH); M + H: magnetic hyperthermia treatment alone group (MNP/MH); F + M: 5FU-MNP treatment group; M; MNP treatment group; F: 5FU treatment group; N: untreated group; GAPDH, glyceraldehyde 3-phosphate dehydrogenase; NF-κB: nuclear factor 'kappa-light-chain-enhancer' of activated B-cells; HSP: heat shock protein. Mean and standard deviation of n = 3.

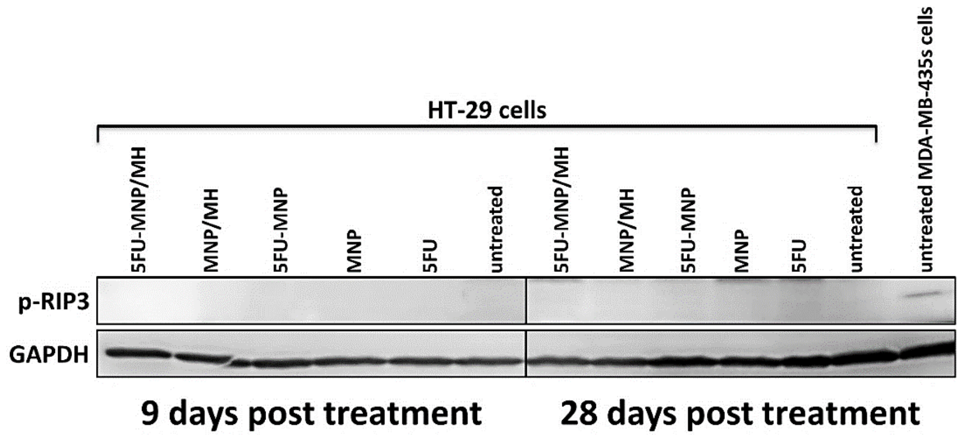

Figure S3. Thermo-chemotherapeutic colon cancer treatment does prospectively not trigger necroptosis. Phosphorylated RIP3 protein that involves in necroptosis did not exist in untreated HT29 cells and was not detectable after the tumor therapies. Purified proteins from untreated MDA-MB-435s human melanoma cells were used as positive control for the expression of phospho-RIP3. GAPDH were used as loading control. MH: magnetic hyperthermia; MNP; magnetic nanoparticle; 5FU: 5-fluorouracil; GAPDH, glyceraldehyde 3-phosphate dehydrogenase; RIP3: receptor-interacting protein 3.

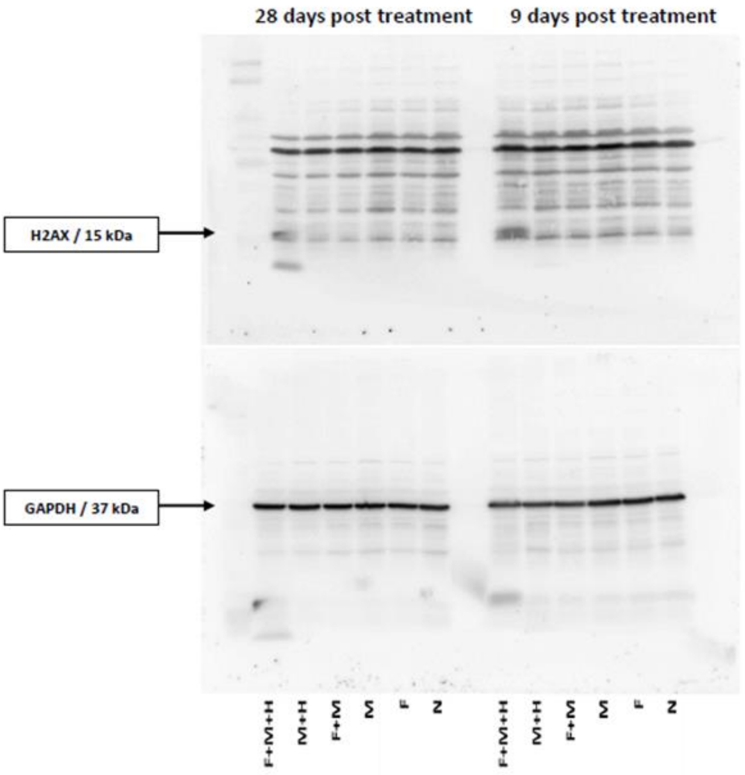
28 days post treatment
9 days post treatment
H2AX / 15 kDa
GAPDH / 37 kDa
F+M+H
M+H
F+M
M
F
N
F+M+H
M+H
F+M
M
F
N

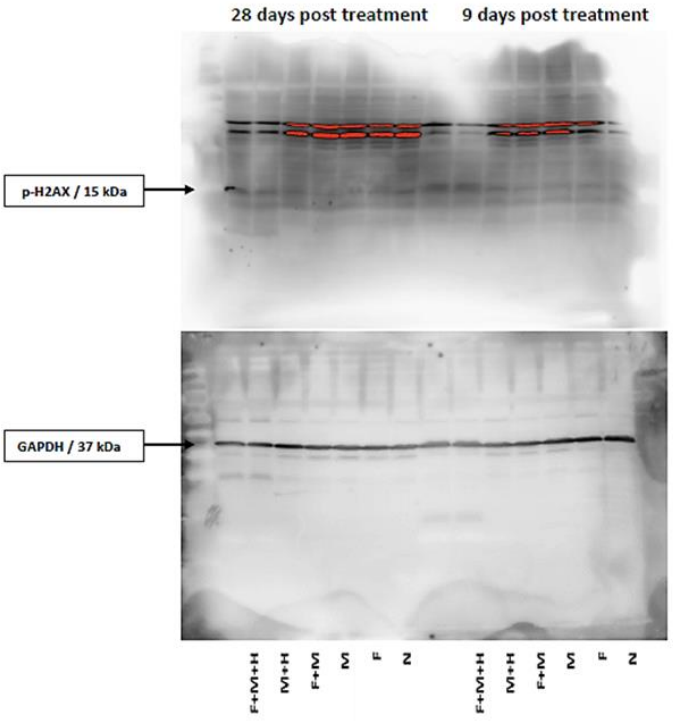
28 days post treatment
9 days post treatment
p-H2AX / 15 kDa
GAPDH / 37 kDa
F+M+H
M+H
F+M
M
F
N
F+M+H
M+H
F+M
M
F
N

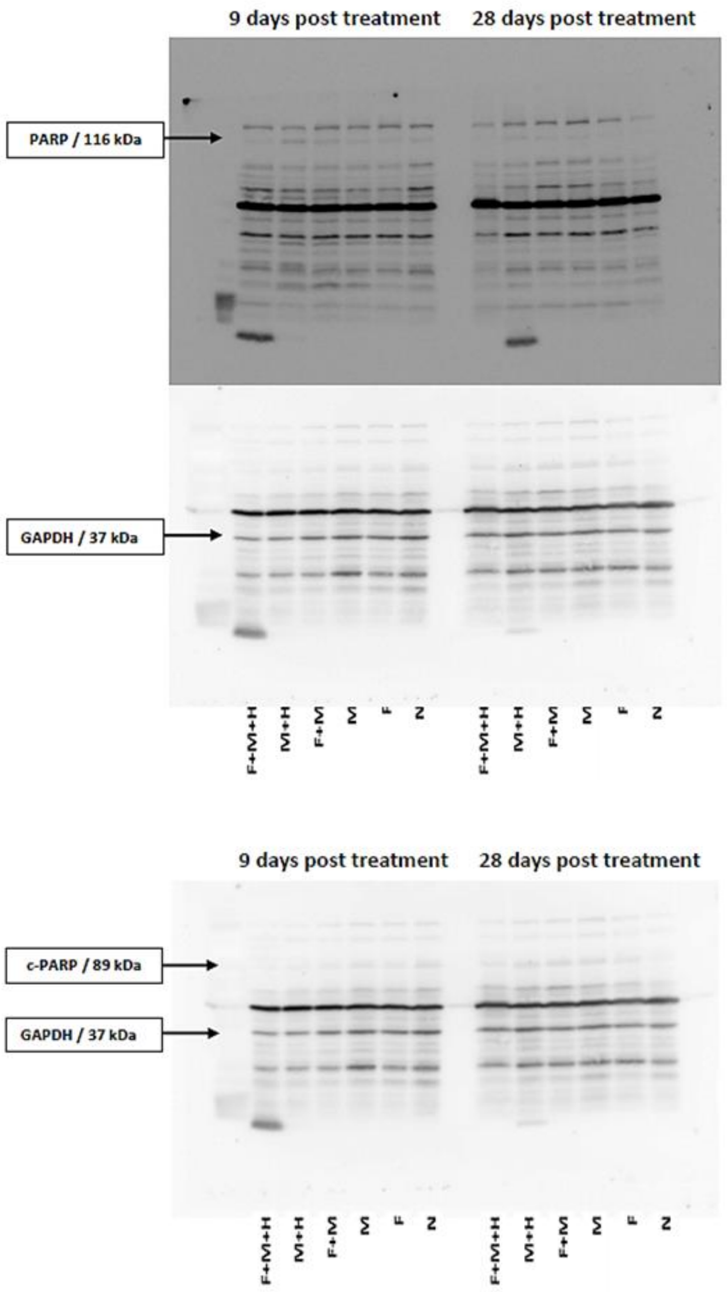

Figure S4. Detail information about Figure 3. F+M+H: combinatorial treatment group (5FU-MNP/MH); M+H: magnetic hyperthermia treatment alone group (MNP/MH); F+M: 5FU-MNP treatment group; M; MNP treatment group; F: 5FU treatment group; N: untreated group.

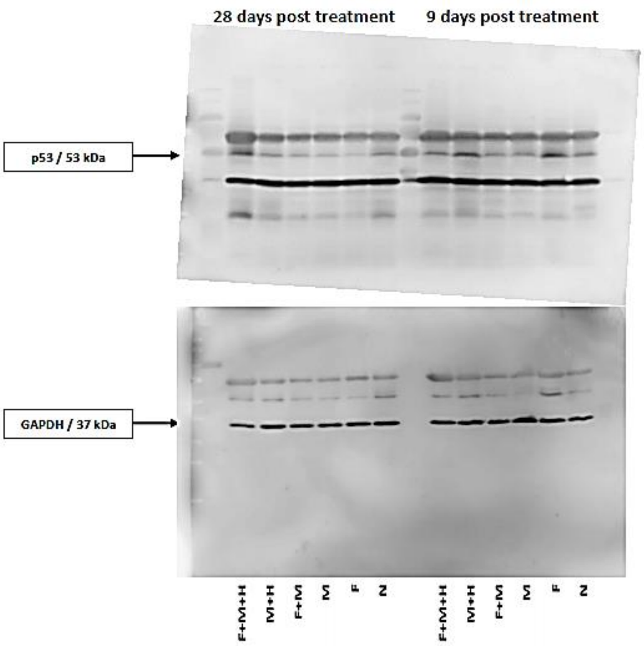
28 days post treatment
9 days post treatment
p53 / 53 kDa
GAPDH / 37 kDa
F+M+H
M+H
F+M
M
F
N
F+M+H
M+H
F+M
M
F
N

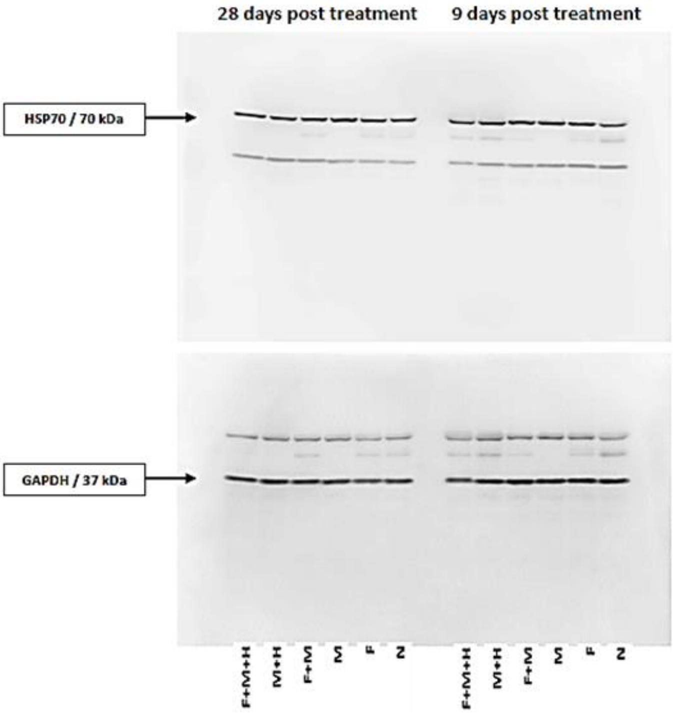
28 days post treatment
9 days post treatment
HSP70 / 70 kDa
GAPDH / 37 kDa
F+M+H
M+H
F+M
M
F
N
F+M+H
M+H
F+M
M
F
N

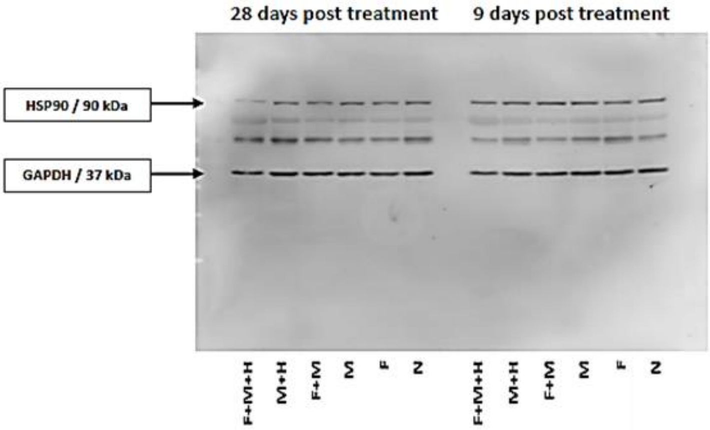
28 days post treatment
9 days post treatment
HSP90 / 90 kDa
GAPDH / 37 kDa
F+M+H
M+H
F+M
M
F
N
F+M+H
M+H
F+M
M
F
N

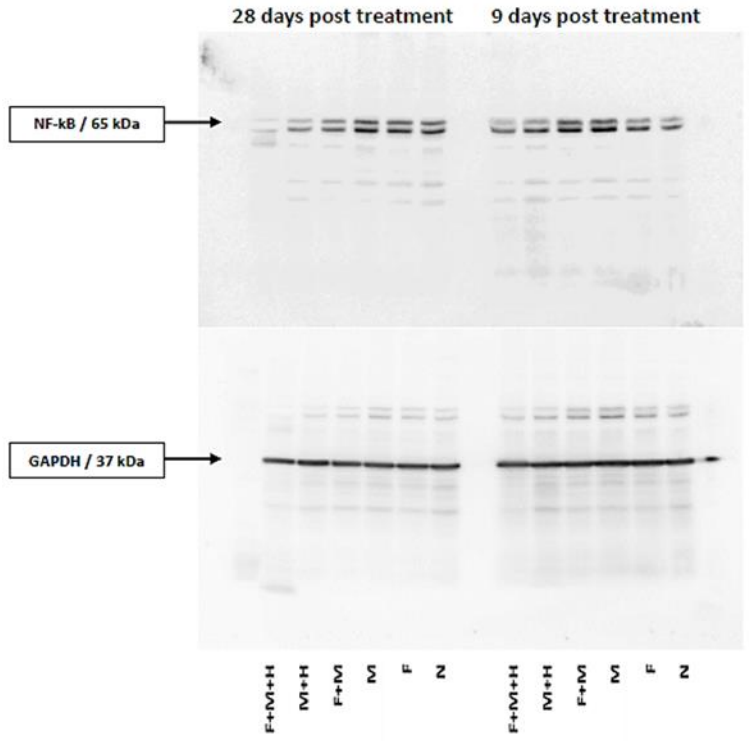

Figure S5. Detail information about Figure 4. F+M+H: combinatorial treatment group (5FU-MNP/MH); M+H: magnetic hyperthermia treatment alone group (MNP/MH); F+M: 5FU-MNP treatment group; M; MNP treatment group; F: 5FU treatment group; N: untreated group.

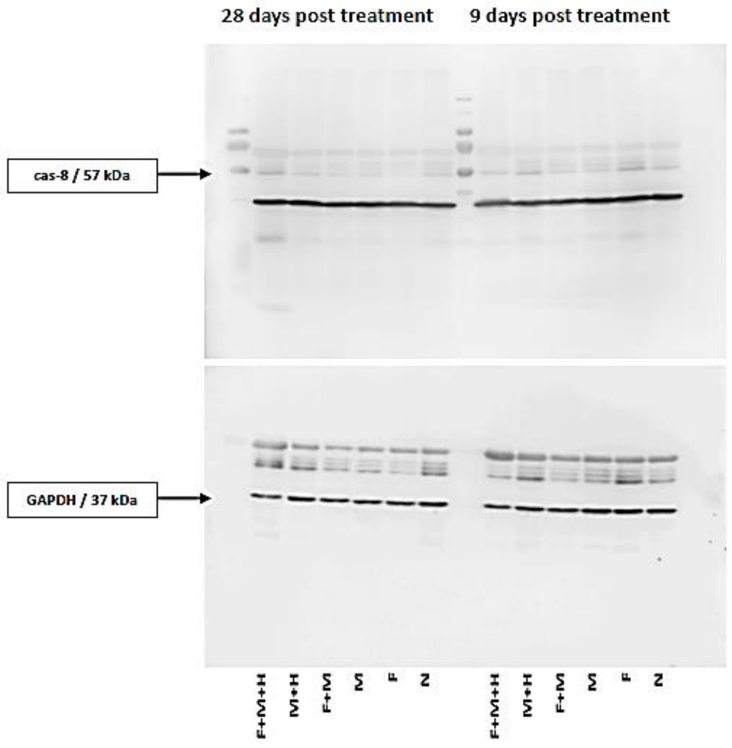

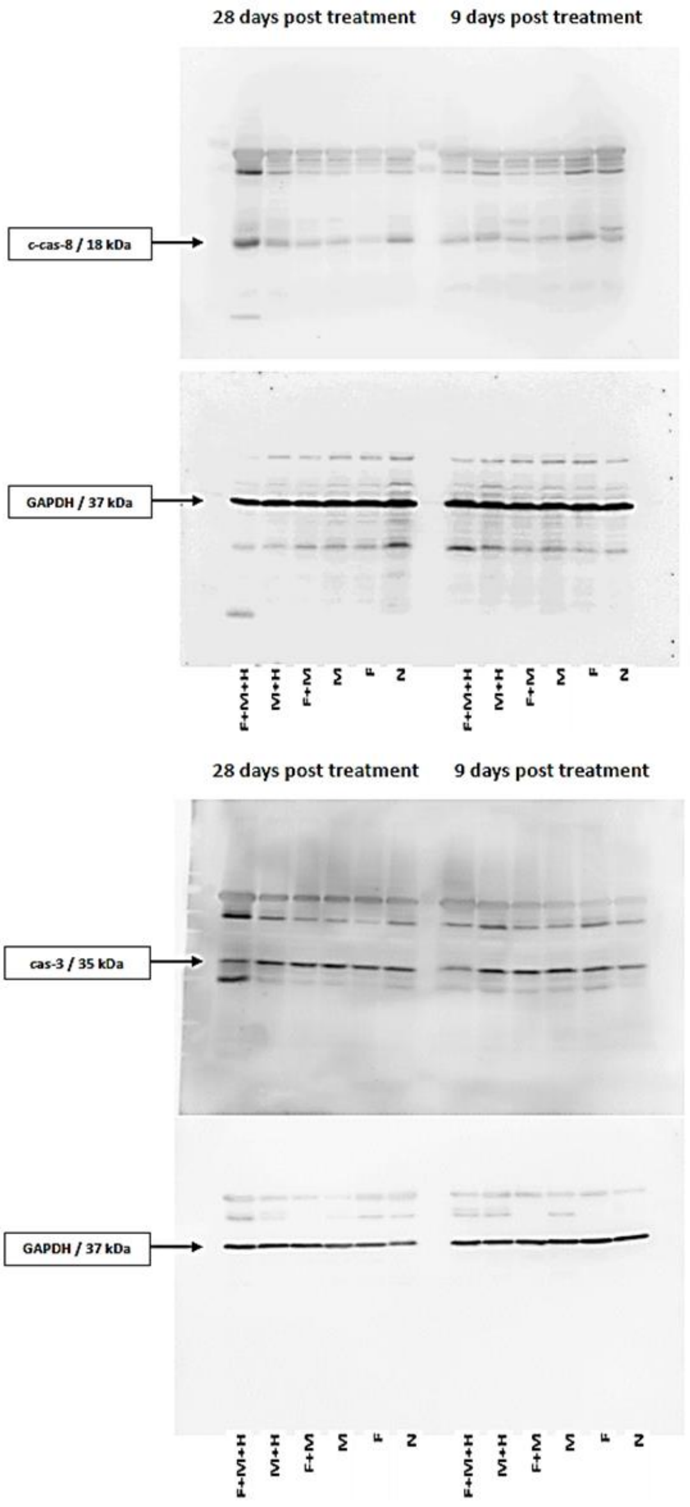

Figure S6. Detail information about Figure S2. F+M+H: combinatorial treatment group (5FU-MNP/MH); M+H: magnetic hyperthermia treatment alone group (MNP/MH); F+M: 5FU-MNP treatment group; M; MNP treatment group; F: 5FU treatment group; N: untreated group.

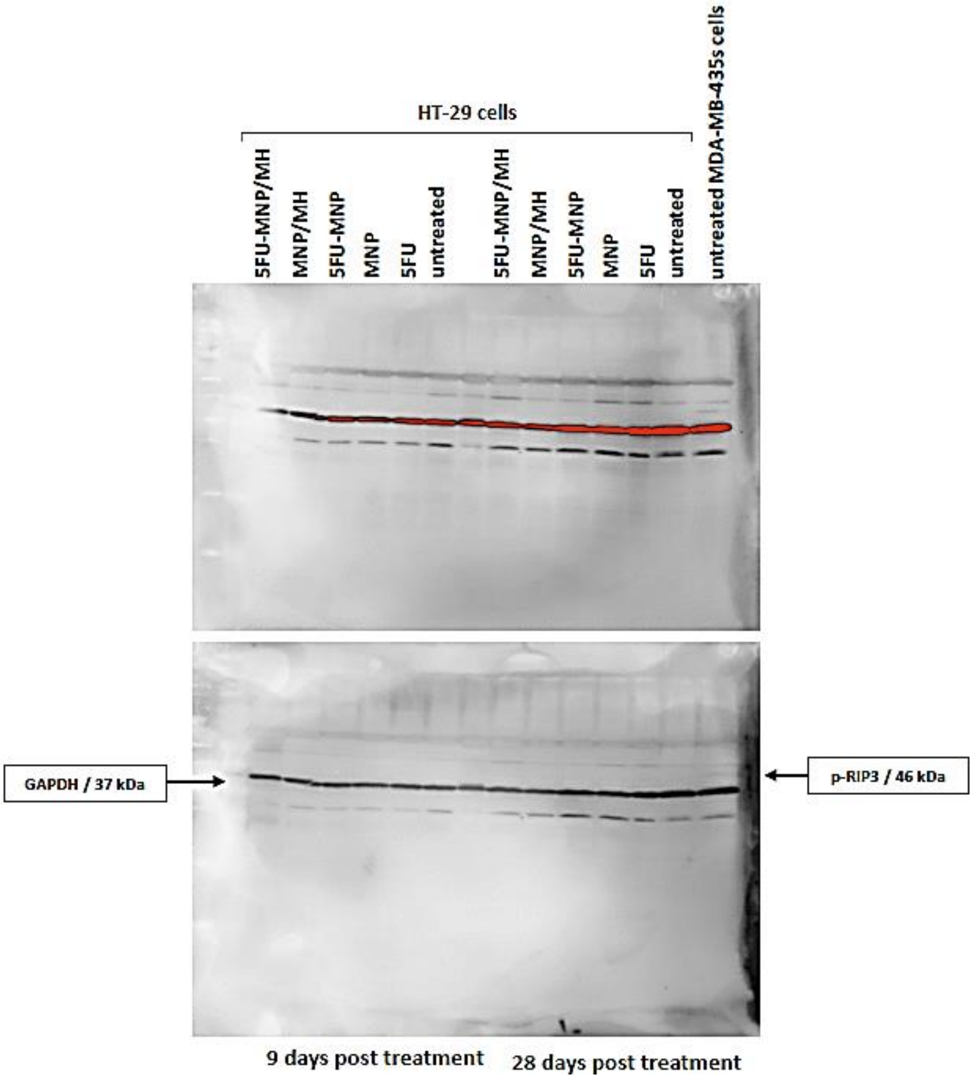

Figure S7. Detail information about Figure S3. MH: magnetic hyperthermia; MNP; magnetic nanoparticle; 5FU: 5-fluorouracil.

© 2020 by the authors. Licensee MDPI, Basel, Switzerland. This article is an open access article distributed under the terms and conditions of the Creative Commons Attribution (CC BY) license (http://creativecommons.org/licenses/by/4.0/).
